# *Helicobacter pylori* virulence genotypes and their relationship with precursor lesions of gastric malignancy and histological parameters in infected patients in Colombia

**DOI:** 10.17843/rpmesp.2023.403.12858

**Published:** 2023-09-26

**Authors:** Claudia Acosta-Astaiza, Alexis López-Sandoval, Juan Bonilla-Chaves, Anyi Valdes-Valdes, William Romo-Romero

**Affiliations:** 1 Research Group in Applied Human Genetics, Universidad del Cauca, Popayán, Colombia. Universidad del Cauca Research Group in Applied Human Genetics Universidad del Cauca Popayán Colombia; 2 Hospital Susana López de Valencia, Popayán, Colombia Hospital Susana López de Valencia Popayán Colombia

**Keywords:** Helicobacter pylori, Gastritis, Bacterial Adhesins, Virulence Factors, Inflammation

## Abstract

The aim of this research was to determine the presence of *Helicobacter pylori* virulence genotypes and their association with precursor lesions of gastric malignancy and histological parameters in patients with dyspepsia symptoms in southwestern Colombia. Polymerase chain reaction (PCR) was used for the genetic characterization of *vacA*, *cagA*, *babA2* and *sabA*. The chi-square or Fischer test were used to evaluate the association between each genotype and the clinical outcome. We found that 86.3% of the patients with precursor lesions of gastric malignancy presented the *vacA* s1/m1 genotype, 68.1% had the *cagA*+ genotype and 68.8% and 55.8% had the *babA2+* and *sabA*+ genotypes, respectively. Our results show association between virulence genotypes and severe degree of polymorphonuclear cell infiltration. In addition, we found an association between the combination of *vacA*/*cagA*, *vacA*/*sabA* and *babA2*/*sabA* genes. This study provides evidence about the association of *H. pylori* virulence genotypes and gastric inflammation in infected patients.

## INTRODUCTION

*Helicobacter pylori* (*H. pylori*) infection is a highly prevalent and significant condition in Colombia; however, not all infected patients develop severe disease such as atrophic gastritis, intestinal metaplasia, or gastric adenocarcinoma ^1^. This variability in disease severity and risk of malignancy suggests that genetic and virulence factors of *H. pylori*, along with host responses, play a critical role in the pathogenesis of these diseases ^1^.

The *vacA* gene of *H. pylori* is highly polymorphic, and its different variants have been linked to different levels of vacuolizing cytotoxin activity, which may influence clinical and histopathological findings in infected patients ^2^^,^^3^. Likewise, the *cagA* gene stands out as another important virulence factor of *H. pylori*, and strains carrying the *cagA*+ genotype are associated with increased gastric mucosal inflammation, cell proliferation and the appearance of gastric precancerous lesions ^4^.

The adhesion genes *babA* and *sabA* play an essential role in the early stages of the infection and inflammation process induced by *H. pylori*. These genes allow the bacteria to adhere to the gastric mucosa, initiating the colonization process. Evidence suggests that these genes are not only involved in the initial colonization, but also play a crucial role in the development of severe disease by participating in the induction of the immune response ^5^. The *babA* gene binds to Lewis B antigens facilitating initial colonization, while the *sabA* gene binds to Sialyl Lewis X antigens, which is relevant for the adherence to the inflamed gastric mucosa ^6^. Like *babA* and *sabA*, the *vacA* and *cagA* genes contribute to the expression and release of interleukin 8 (IL-8), macrophage inflammatory protein (MIP)-1α and (MIP)-1β by neutrophils, these *H. pylori* virulence factors are associated with increased polymorphonuclear neutrophil infiltration in areas of intestinal atrophy and metaplasia ^7^.

Understanding the relationship between the genetic diversity of *H. pylori* and the host immune response in the context of virulence genes is essential to unveil the molecular basis of gastric diseases associated with this bacterium. This is fundamental to develop more precise and effective prevention and treatment strategies. Therefore, this study aimed to investigate the association between the variability of the virulence genes *cagA*, *vacA*, *babA* and *sabA* of *H. pylori* and gastric malignancy precursor lesions as well as histological parameters in Colombian patients.

**KEY MESSAGES**

Motivation for the study. The high local prevalence of *H. pylori* and its genetic diversity are key factors for the development of digestive diseases. Identifying the virulent genotypes of *H. pylori* and their association with gastric precursor lesions, as well as histological parameters, could improve the understanding of its pathogenesis and severity of related diseases.

Main findings. The *vacA* s1/m1 genotype was more frequent in subjects with gastric precursor lesions. High polymorphonuclear cell infiltration is associated with *vacA* s1/m1, *cagA*+, *babA*2+, *sabA*+.

Implications. It is suggested that *H. pylori* genotypes influence gastric inflammation, contributing to the early prognosis of gastric preneoplastic lesions.

## THE STUDY

### Study population

A descriptive cross-sectional study was conducted in 290 patients who attended the outpatient service of the Gastroenterology Unit from the Susana López de Valencia Hospital in the city of Popayán (located in southwestern Colombia), between February and December 2022. The sample was selected by convenience and included patients older than 18 years with dyspepsia symptoms and who were referred to gastric endoscopy during the last year. Patients with previous treatment of *H. pylori* infection with antibiotics or bismuth salts were excluded. Participants were divided into two groups: those with chronic gastritis and those with precursor lesions of gastric malignancy (atrophic gastritis and intestinal metaplasia).

### Gastric biopsies and histopathology

We obtained five samples, two biopsies of the antrum (greater and lesser curvature), two of the body (greater and lesser curvature) and one biopsy of the angular incisure. The biopsies were fixed in buffered formalin and stained with hematoxylin-eosin and Giemsa stains. An experienced pathologist carried out the histopathological analysis of each specimen. Analogous visual scales were used according to the Sydney system ^8^. We assessed the presence of inflammatory activity (inflammation by polymorphonuclear neutrophils) as well as the presence of chronic gastritis, atrophic gastritis and intestinal metaplasia. Additionally, two antrum and body samples were obtained for molecular analysis of *H. pylori*.

### Detection of *H. pylori vacA, cagA, babA2* and *sabA* genes

Bacterial genotyping was carried out by extracting DNA from biopsies using the Wizard Genomic DNA Purification extraction kit (Promega®, Madison, WI, USA) and following the manufacturer’s recommendations. The extracted DNA samples were stored at -30 °C until use. DNA quality was determined by A260/A280 absorbance ratio using a Thermo Scientific® NanoDrop 2000 TM spectrophotometer. The tests were carried out by biologists at the Human Genetics Laboratory of the Universidad del Cauca.

We used the QIAGEN Multiplex PCR® kit for gene amplification utilizing the following primers: *vacA* (VAI-F- ATGGAAATACAACAACAAACACACACAC; VAI-R- CTGCTTGAATGCGCGCCAAAC; VAG-F-CAATCTGTCCAATCAAGCGAG; VAG-R- GCGTCAAAATAATTCCAAGG) and *cagA* (cagA-F-GATAACAGGCAAGGCAAGCTTTTGAGG; cagA-R- CTGCAAAAGATTGTTGTTTGGCAGA); the amplification conditions were standardized as suggested by the kit manufacturer. The *babA2* (babA2-F- CCAAACGAAACGAAACAAAAAAAGCGT; babA2-R-GCTTGTGTAAAAGCCGTCGT) and *sabA* (sabA-F- TTTTTTTGTCAGCTACGCGTTC; sabA-R-ACCGAAGTGATAACGGCTTG) genes were evaluated by conventional polymerase chain reaction (PCR) as suggested by Yadegar *et al*. ^9^. *H. pylori* strains NCTC-11637, NCTC-11638, and clinical isolate 3062 were provided by the National Cancer Institute as positive controls. Amplification products were stained with 3 μL of EZ-vision (AMRESCO®) and analyzed by 1.5% agarose gel electrophoresis at 80 volts for one hour, visualized by UV transilluminator with a wavelength of 254/365 nm and compared with positive and negative controls. Pilot tests were carried out to verify the quality of the reagents and extraction kits, they also verified that the laboratory equipment had been previously calibrated.

### Dependent and independent variable

The dependent variables were the precursor lesions of gastric malignancy, which includes atrophic gastritis and intestinal metaplasia, and histological parameters, such as the degree of polymorphonuclear cell infiltration and the degree of atrophy. Each of these histological variables was categorized into four severity levels: none, mild, moderate and severe. The independent variables were the *H. pylori* virulence genotypes, which included *vacA*, *cagA*, *babA* and *sabA*, and were measured as being present (+) or absent (-).

### Covariates

We included other sociodemographic variables such as age in years, which was categorized into three groups (18-40 years, 41-60 years and ≥ 61 years); sex (men and women); income as the minimum living wage (MLW) (where < 1 MLW stands for less than a living wage and ≥ 1 MLW stands for greater than or equal to a living wage); place of origin (urban and rural); educational level of patients (none, primary, secondary and higher education).

### Statistical analysis

Categorical variables were tabulated by using absolute and relative frequencies. The chi-square or Fisher test were used to compare *H. pylori* virulence genotypes with precursor lesions of gastric malignancy and histologic parameters. The SPSS version 25 software was used. Categorical variables are presented as proportions and absolute frequencies. Cramer’s correlation coefficient was used to determine the degree of association between genotypes. A value of p<0.05 was considered to be significant.

### Ethical Aspects

Participants accepted to take part in the study and signed the informed consent form. This research was approved by the Ethics Committee for Scientific Research of the Universidad del Cauca. Act 6.1-1.25/5 of January 23, 2022.

## FINDINGS

### Population characteristics

We included 290 patients. Among the main demographic characteristics, we found that most participants were in the age group between 41 and 60 years, representing 45.2% of the sample. In addition, there was a marked predominance of the female gender, which constituted 73.8% of the participants. It is important to mention that 87.9% of the population had monthly incomes below the minimum wage. Regarding their geographic origin, 61.7% of the patients lived in rural areas. Most participants had only primary education (57.2%) (Table 1).

**Table 1:** Distribution of sociodemographic characteristics of the studied population.

Characteristics	Total n=290 (%)
Age	
18-40 years	88 (30.3)
41-60 years	131 (45.2)
≥ 61 years	71 (24.5)
Sex	
Men	76 (26.2)
Women	214 (73.8)
Income	
<1 MLW	255 (87.9)
≥1 MLW	35 (12.1)
Residence	
Urban	111 (38.3)
Rural	179 (61.7)
Education level	
None	6 (2.1)
Primary school	166 (57.2)
Secondary school	70 (24.1)
Higher education	48 (16.6)

MLW: minimum living wage; < 1 MLW: less than a minimum living wage; ≥ 1

MLW: greater than or equal to a minimum living wage.

### *vacA, cagA, babA* and *sabA* genotypes and precursor lesions of gastric malignancy

Our results show 217 participants who were positive for *H. pylori* by molecular diagnosis, of which 63.6% had precursor lesions of gastric malignancy (PLGM) and 36.4% had chronic gastritis (CG). The *vacA* s1/m1 and *cagA*+ virulence genotypes were more frequent in patients with PLGM with 86.3% and 68.1%, respectively. The *vacA* s2/m2 genotype was more frequent in patients with chronic gastritis. The frequency of *babA2*+ and *sabA*+ genotypes was higher in the population with PLGM with 68.8% and 55.8%, respectively; although there were no significant differences between patients with CG and patients with PLGM (p>0.05) (Table 2). Additionally, we evaluated the degree of association of the genes with each other. There was a significant correlation between the presence of the *vacA* gene and the *cagA* and *sabA* genes with a medium degree of association (*VvacA*/*cagA*= 0.359, *VvacA*/*sabA*=0.332), as well as between the *sabA*/*babA2* genes (*VsabA*/*babA2*=0.506); however, no correlation was found between the other genes (Table 3).

**Table 2:** Relationship of *H. pylori cagA, vacA, babA*2 and *sabA* genotypes with precursor lesions of gastric malignancy.

Genotype	Total n=217 n (%)	CG n=79 n (%)	PLGM n=138 n (%)	p-value ^a^
*cagA -*	78 (35.9)	34 (43)	44 (31.9)	0.099
*cagA +*	139 (64.1)	45 (57)	94 (68.1)	
*babA -*	60 (27.6)	17 (21.5)	43 (31.2)	0.127
*babA +*	157 (72.4)	62 (78.5)	95 (68.8)	
*sabA -*	93 (42.9)	32 (40.5)	61 (44.2)	0.596
*sabA +*	124 (57.1)	47 (59.5)	77 (55.8)	
*vacA*				
*s1/m1*	177 (81.6)	58 (73.4)	119 (86.3)	0.110
*s1/m2*	8 (3.7)	4 (5.1)	4 (2.9)	
*s2/m2*	15 (6.9)	9 (11.4)	6 (4.3)	
Coinfection	17 (7.8)	8 (10.1)	9 (6.5)	

a p-value calculated with the chi-square test.

CG: chronic gastritis. PLGM: precursor lesions of gastric malignancy.

**Table 3:** Relationship between *H. pylori* genotypes.

Genotypes	Degree of association ^a^	p-value
*cagA-babA*	0.138	0.428 ^b^
*cagA-sabA*	0.128	0.601 ^b^
*cagA-vacA*	0.359	0.001 ^c^
*sabA-babA*	0.506	0.001 ^b^
*sabA-vacA*	0.332	0.001 ^c^
*babA-vacA*	0.177	0.152 ^c^

a Degree of association calculated with Cramer’s correlation coefficient.

b p-value calculated with the chi-square test.

c p-value calculated with Fisher’s exact test.

### *vacA, cagA, babA* and *sabA* genotypes and histological parameters

Considering the importance of the different *H. pylori* virulence genotypes on histological parameters, we determined that the allelic combination of the *vacA* s1/m1 gene, the *cagA*+, *babA2*+ and *sabA*+ genotypes are closely related to inflammatory activity, paarticularly to the severe degree of polymorphonuclear cell infiltration (p<0.05). In addition, we found a significant relationship between the presence of the *cagA*+ genotype and the occurrence of gastric atrophy in its most severe form (p<0.05). Other genotypes showed no relationship with the degree of atrophy (Table 4).

**Table 4:** Relationship between *H. pylori* virulence genotypes and histological parameters.

Parameters	Polymorphonuclear cell infiltration					Atrophy				
	None n=52 n (%)	Mild n=41 n (%)	Moderate n=64 n (%)	Severe n=60 n (%)	p-value	None n=81 n (%)	Mild n=51 n (%)	Moderate n=59 n (%)	Severe n=26 n (%)	p-value
*cagA-*	29 (55.8)	14 (34.1)	9 (14.1)	26 (43.3)	0.001 ^a^	34 (42.0)	8 (15.7)	24 (40.7)	12 (46.2)	0.007 ^a^
*cagA+*	23 (44.2)	27 (65.9)	55 (85.9)	34 (56.7)		47 (58.0)	43 (84.3)	35 (59.3)	14 (53.8)	
*babA2-*	43 (82.7)	4 (9.8)	6 (9.4)	7 (11.7)	0.001 ^a^	18 (22.2)	15 (29.4)	23 (39)	4 (15.4)	0.071 ^a^
*babA2+*	9 (17.3)	37 (90.2)	58 (90.6)	53 (88.3)		63 (77.8)	36 (70.6)	36 (61)	22 (84.6)	
*sabA-*	49 (94.2)	5 (12.2)	14 (21.9)	25 (41.7)	0.001 ^a^	33 (40.7)	19 (37.3)	33 (55.9)	8 (30.8)	0.091 ^a^
*sabA+*	3 (5.8)	36 (87.8)	50 (78.1)	35 (58.3)		48 (59.3)	32 (62.7)	26 (44.1)	18 (69.2)	
s1/m1	40 (76.9)	28 (68.3)	58 (90.6)	51 (85)	0.001 ^b^	60 (74.1)	44 (86.3)	53 (89.8)	20 (76.9)	0.055 ^b^
s1/m2	0 (0.0)	4 (9.8)	1 (1.6)	3 (5)		4 (4.9)	1 (2.0)	1 (1.7)	2 (7.7)	
Coinfection	11 (21.2)	1 (2.4)	1 (1.6)	4 (6.7)		8 (9.9)	1 (2.0)	4 (6.8)	4 (15.4)	
s2/m2	1 (1.9)	8 (19.5)	4 (6.2)	2 (3.3)		9 (11.1)	5 (9.7)	1 (1.7)	0	

a p-value calculated with the chi-square test.

b p-value calculated with Fisher’s exact test.

## DISCUSSION

In this study we analyzed the presence of *Helicobacter pylori vacA*, *cagA*, *babA* and *sabA* genotypes and their association with gastric malignancy precursor lesions and histological parameters in infected patients from southwestern Colombia. We found that the s1/m1 genotypes of the *vacA* and *cagA*+ gene were more frequent in patients with PLGM, similar to previous studies in Colombia ^10^^,^^11^. This finding suggests that the studied population has a high frequency of more virulent bacterial strains that could increase the risk of gastrointestinal diseases. The distribution of s1 and m1 alleles has been found to be associated with diseases such as atrophic gastritis, intestinal metaplasia, and high risk of gastric cancer ^12^. We found a relationship between the *vacA* virulence genes and the *cagA* and *sabA* genes, suggesting that these genes may act synergistically to increase gastric inflammation and the risk of developing PLGM. Our results show an increased frequency of *babA2*+ and *sabA*+ genotypes in PLGM, although there was no significant association. However, some studies in other countries have described that *babA2*+ and *sabA*+ strains are associated with increased risk of developing intestinal atrophy, intestinal metaplasia, and gastric cancer ^13^^,^^14^. Differences between populations may be due to the small sample size, heterogeneity between studies, and geographic factors.

Ours is the latest study to explore the relationship between *H. pylori* virulence genotypes and histological parameters in a Colombian population. A previous Colombian study reported that strains with *vacA* s1 and m1 genotypes were closely associated with changes in gastric histology, while neutrophil infiltration, intestinal metaplasia and atrophy are significantly higher in *babA2* gene positive cases compared to babA2 negative cases ^15^. Studies from different countries have reported that inflammatory activity is more prominent in patients infected with strains harboring all virulent variants of *H. pylori* (*vacA* s1m1/*cagA*+/*babA2*+) ^16^. Also, colonization with virulence factors such as *cagA*+, *iceA1*-, and *oipA*+ has been found to correlate significantly with high polymorphonuclear cell infiltration, whereas colonization with *cagA*+, *babA2*+, and *oipA*+ is strongly related to higher mononuclear cell infiltration scores ^1^. The above findings are consistent with what was found by our study.

*H. pylori* infection causes local inflammation in the gastric mucosa due to migration and infiltration of immune cells, leading to disease progression. The *cagA* and *vacA* proteins produced by *H. pylori* stimulate a proinflammatory response leading to gastric inflammation. IL-8 production attracts immune cells to infiltrate the infection site, which favors a persistent inflammatory state ^7^^,^^17^. This may explain why the positive *cagA* and *vacA* status were related to polymorphonuclear cell infiltration in our study. The *sabA*+ genotype is also related to the induction of the inflammatory response leading to the development of gastric diseases. *H. pylori sabA*-positive strains stimulate neutrophil activation causing gastric epithelial damage ^5^. Our results show high polymorphonuclear cell infiltration in patients with the *sabA*+ genotype. These findings are consistent with other studies that show a relationship between *sabA* expression and gastric inflammation ^18^.

By analyzing the adhesion genes *babA* and *sabA*, as well as the *vacA* and *cagA* genes, we can expand the evidence on their role in initial colonization, induction of immune responses and subsequent progression of gastric lesions. This research will contribute to fill the knowledge gap in the Colombian population and may have a significant impact on public health by identifying subgroups of patients at higher risk of developing severe gastric disease. The results of this study could provide valuable information to guide prevention strategies, early diagnosis and effective treatment in patients with *H. pylori* infection in Colombia and, possibly, in other populations with similar genetic characteristics.

One of the limitations of our study is the small number of patients included. In addition, we did not analyze other virulence factors of *H. pylori* that could be playing a role in mechanisms associated with the development of precursor lesions of gastric malignancy.

In conclusion, our findings show a high frequency of *H. pylori vacA* s1/m1 genotype in a Colombian population and the cytotoxic activity of this genotype could be enhanced by other *H. pylori* bacterial factors such as the presence of *cagA*+ and *sabA*+ genotypes. The *vacA* s1/m1 allelic combination is related to other *H. pylori* bacterial factors such as the presence of *cagA*+ and *sabA*+ genotypes. This study shows a relationship between the studied virulence genotypes and the degree of infiltration of polymorphonuclear cells, which play an important role in inflammatory activity, which could increase the progression of gastric malignancy precursor lesions. Further complementary studies, including negative *H. pylori* controls, are needed to strengthen this hypothesis.
