# Supplementary material for: Helicobacter pylori virulence genotypes and their relationship with precursor lesions of gastric malignancy and histological parameters in infected patients in Colombia
Source: Rev Peru Med Exp Salud Publica. 2023 Sep 26;40(3):348–53. doi: 10.17843/rpmesp.2023.403.12858 (PMC10953661; doi:10.17843/rpmesp.2023.403.12858)

**Material complementario (imágenes de histopatología).**

**GC (Gastritis crónica)**

**Descripción:** mucosa del cuerpo con moderado infiltrado inflamatorio mononuclear y preservación de la densidad glandular compatible con gastritis crónica no atrófica (10X).


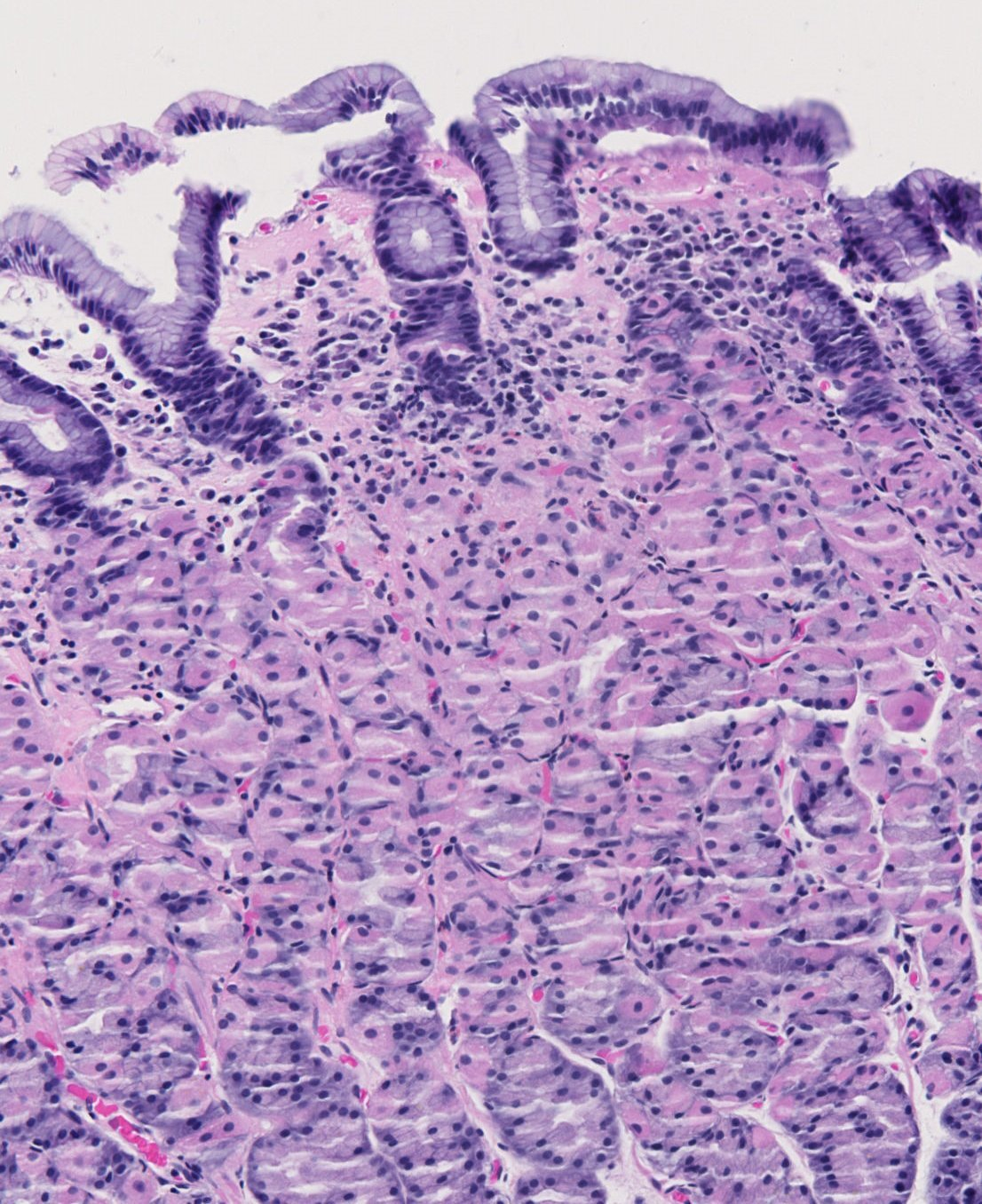


**GCA (Gastritis crónica atrófica)**

**Descripción:** Se identifica mucosa gástrica con moderado infiltrado inflamatorio mononuclear asociado a disminución de la densidad glandular. La lámina propia exhibe fibrosis compatible con gastritis crónica atrófica (20X).


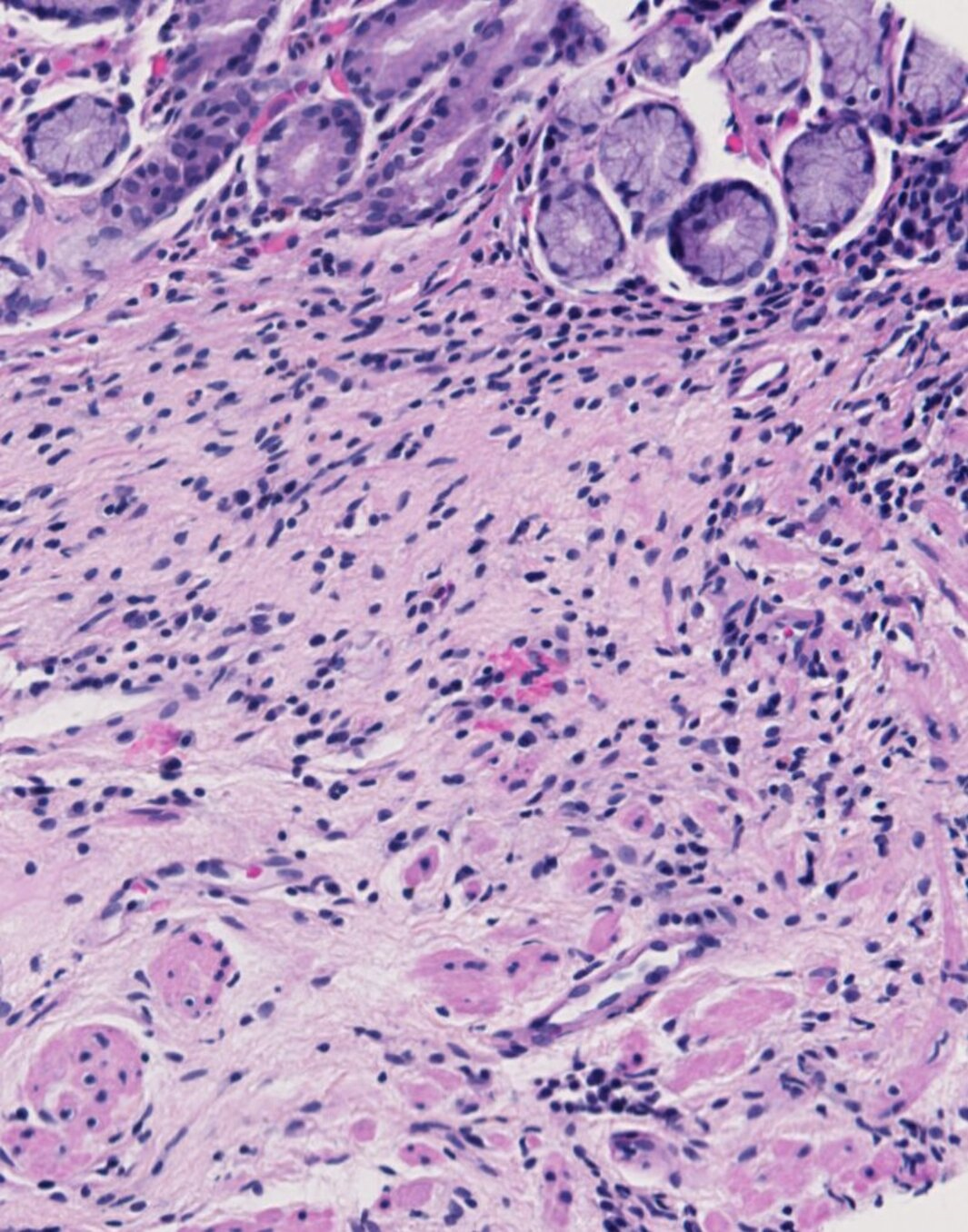


**MI (Metaplasia Intestinal)**

**Descripción:** Mucosa gástrica antral con disminución de la densidad glandular y sustitución del epitelio por células caliciformes. Hallazgos compatibles con metaplasia intestinal (20X).


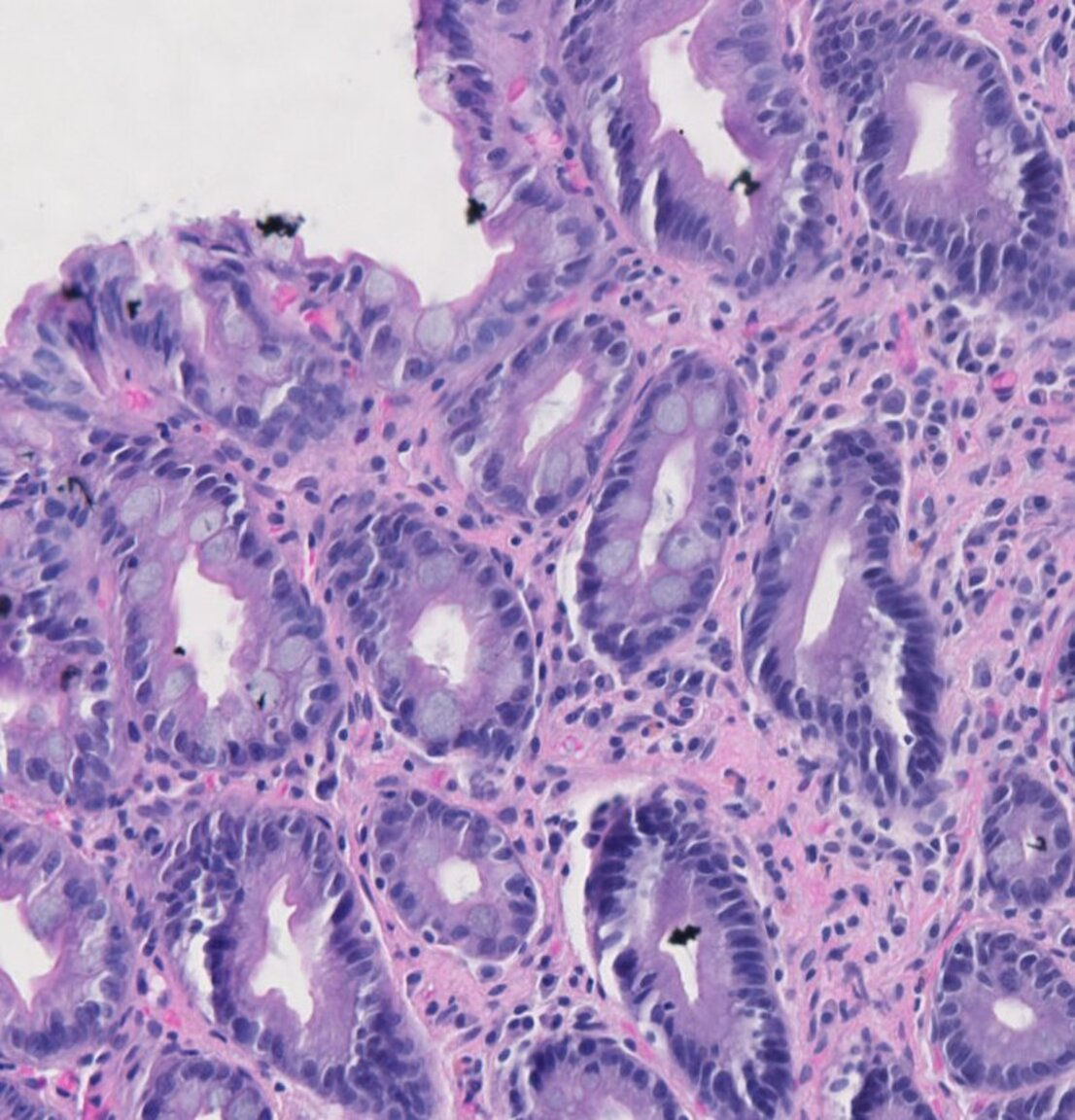

Supplement: Supplementary material. — Available in the electronic version of the RPMESP. [file rpmesp-40-03-12858-s001.docx]
