# Supplementary material for: Helicobacter pylori virulence genotypes and their relationship with precursor lesions of gastric malignancy and histological parameters in infected patients in Colombia
Source: Rev Peru Med Exp Salud Publica. 2023 Sep 26;40(3):348–53. doi: 10.17843/rpmesp.2023.403.12858 (PMC10953661; doi:10.17843/rpmesp.2023.403.12858)
Supplement: Translation

# Translation: Genotipos de virulencia de *Helicobacter pylori* y su asociación con lesiones precursoras de malignidad gástrica y parámetros histológicos en pacientes colombianos

**Authors:** Claudia Acosta-Astaiza, Alexis López-Sandoval, Juan Bonilla-Chaves, Anyi Valdes-Valdes, William Romo-Romero

**Affiliations:** 1 Grupo de Investigación en Genética Humana Aplicada, Universidad del Cauca, Popayán, Colombia.; 2 Hospital Susana López de Valencia, Popayán, Colombia.

**Keywords:** Helicobacter pylori, Gastritis, Adhesinas Bacterianas, Factores de Virulencia, Inflamación

## Abstract

Se determinó la presencia de los genotipos de virulencia de *Helicobacter pylori* y su asociación con las lesiones precursoras de malignidad gástrica y parámetros histológicos en pacientes con síntomas de dispepsia del suroccidente de Colombia. Se realizó reacción en cadena de polimerasa (PCR) para la caracterización genética de *vacA*, *cagA*, *babA2* y *sabA*. Se empleó la prueba de chi cuadrado o Fischer para evaluar la asociación de cada genotipo sobre el desenlace clínico. En los pacientes con lesiones precursoras de malignidad gástrica se encontró que el 86,3% presentaron el genotipo *vacA* s1/m1, el 68,1% *cagA+* y los genotipos *babA2+* y *sabA+* con el 68,8% y 55,8%, respectivamente. También, se demostró la asociación entre los genotipos de virulencia y el grado severo de infiltración de células polimorfonucleares. Además, se encontró una asociación entre la combinación de los genes *vacA*/*cagA*, *vacA*/*sabA* y *babA2*/*sabA*. Este estudio proporciona evidencia acerca de la asociación de los genotipos de virulencia del *H. pylori* y la inflamación gástrica en pacientes infectados.

## INTRODUCCIÓN

La infección por *Helicobacter pylori* (*H. pylori*) es una condición altamente prevalente y significativa en Colombia; sin embargo, no todos los pacientes infectados desarrollan enfermedades graves como gastritis atrófica, metaplasia intestinal y adenocarcinoma gástrico ^1^. Esta variabilidad en la gravedad de la enfermedad y el riesgo de malignidad sugiere que factores genéticos y de virulencia de *H. pylori*, junto con las respuestas del huésped, desempeñan un papel crítico en la patogénesis de estas enfermedades ^1^.

El gen *vacA* de *H. pylori* es altamente polimórfico, y sus diferentes variantes se han vinculado con distintos niveles de actividad de citotoxina vacuolizante, lo que puede influir en los resultados clínicos e histopatológicos en pacientes infectados ^2^^,^^3^. Asimismo, el gen *cagA* se destaca como otro factor importante de virulencia de *H. pylori*, y las cepas que portan el genotipo *cagA*+ están relacionadas con un incremento de la inflamación de la mucosa gástrica, la proliferación celular y la aparición de lesiones precancerosas gástricas ^4^.

Los genes de adhesión *babA* y *sabA*, desempeñan un papel esencial en las primeras etapas del proceso de infección e inflamación inducido por *H. pylori*. Estos genes permiten que la bacteria se adhiera a la mucosa gástrica, dando inicio al proceso de colonización. La evidencia sugiere que estos genes no sólo están implicados en la colonización inicial, sino que también desempeñan una función crucial en el desarrollo de enfermedades graves al participar en la inducción de la respuesta inmunológica ^5^. El gen *babA*, se une a los antígenos de Lewis B, facilitando la colonización inicial, mientras que el gen *sabA* se une a los antígenos Sialil Lewis X, lo que es relevante en el proceso de adherencia a la mucosa gástrica inflamada ^6^. Al igual que *babA* y *sabA*, los genes *vacA* y *cagA* contribuyen a la expresión y liberación de interleuquina 8 (IL-8), proteína inflamatoria de macrófagos (MIP)-1α y (MIP)-1β por parte de los neutrófilos, estos factores de virulencia de *H. pylori* están asociados con una mayor infiltración de neutrófilos polimorfonucleares en áreas de atrofia y metaplasia intestinal ^7^.

La comprensión de la relación entre la diversidad genética de *H. pylori* y la respuesta inmunológica del huésped en el contexto de los genes de virulencia es esencial para develar las bases moleculares de las enfermedades gástricas asociadas con esta bacteria. Esto es fundamental para desarrollar estrategias de prevención y tratamiento más precisas y efectivas. En este sentido, en el presente estudio se propone investigar la asociación entre la variabilidad de los genes de virulencia *cagA, vacA, babA y sabA* de *H. pylori* con las lesiones precursoras de malignidad gástrica y los parámetros histológicos en pacientes colombianos.

**MENSAJES CLAVE**

Motivación para realizar el estudio. La alta prevalencia local de *H. pylori* y su diversidad genética son factores clave para el desarrollo de patologías gástricas. Conocer los genotipos virulentos de *H. pylori* y su asociación con lesiones precursoras gástricas, así como parámetros histológicos, podría mejorar la comprensión de su patogenia y gravedad de las enfermedades relacionadas.

Principales hallazgos. El genotipo *vacA* s1/m1 fue mayor en sujetos con lesiones precursoras gástricas. La alta infiltración de células polimorfonucleares se asocia con *vacA* s1/m1, *cagA*+, *babA*2+, *sabA*+.

Implicancias. Se sugiere que los genotipos de *H. pylori* influyen en la inflamación gástrica, aportando al pronóstico temprano de lesiones preneoplásicas gástricas.

## EL ESTUDIO

### Población de estudio

Se realizó un estudio descriptivo de corte transversal en 290 pacientes que asistieron por consulta externa a la Unidad de Gastroenterología del Hospital Susana López de Valencia de la ciudad de Popayán (ubicada en el suroccidente de Colombia), entre febrero y diciembre del 2022. La muestra se seleccionó por conveniencia e incluyó pacientes mayores de 18 años con síntomas de dispepsia y remitidos a endoscopia gástrica durante el último año. Se excluyeron pacientes con tratamiento previo de infección para *H. pylori* con antibióticos o sales de bismuto*.* Los pacientes se dividieron en dos grupos: con gastritis crónica y con lesiones precursoras de malignidad gástrica (gastritis atrófica y metaplasia intestinal).

### Toma de biopsias gástricas e histopatología

Se tomaron cinco muestras correspondientes a dos biopsias de antro (curvatura mayor y menor), dos de cuerpo (curvatura mayor y menor) y una biopsia de incisura *angularis*. Las biopsias se fijaron en formol tamponado y fueron coloreadas con las tinciones de hematoxilina-eosina y Giemsa. El análisis histopatológico de cada muestra lo realizó un patólogo experimentado. Se emplearon escalas visuales análogas de acuerdo al sistema de Sydney ^8^. Se evaluó la presencia de actividad inflamatoria (inflamación por neutrófilos polimorfonucleares) y la presencia de gastritis crónica, gastritis atrófica y metaplasia intestinal. Adicionalmente, fueron tomadas dos muestras de antro y cuerpo para análisis molecular de *H. pylori*.

### Detección de los genes *vacA, cagA, babA2 y sabA* de *H. pylori*

Los estudios de genotipificación bacteriana fueron realizados a partir de la extracción del ADN de las biopsias, utilizando el kit de extracción Wizard Genomic DNA Purification (Promega®, Madison, WI, USA) y siguiendo las recomendaciones del fabricante. Las muestras de ADN extraídas fueron almacenadas a -30 °C hasta su uso. La calidad del ADN se determinó por medio de la relación de absorbancia A260/A280 usando un espectrofotómetro NanoDrop 2000 ^TM^ Thermo Scientific®. Las pruebas fueron llevadas a cabo por biólogos en el Laboratorio de Genética Humana de la Universidad del Cauca.

Para la amplificación de los genes, se utilizó el kit QIAGEN Multiplex PCR® utilizando los siguientes cebadores: *vacA* (VAI-F- ATGGAAATACAACAAACACAC; VAI-R- CTGCTTGAATGCGCCAAAC; VAG-F-CAATCTGTCCAATCAAGCGAG; VAG-R- GCGTCAAAATAATTCCAAGG) y *cagA* (cagA-F-GATAACAGGCAAGCTTTTGAGG; cagA-R- CTGCAAAAGATTGTTTGGCAGA), las condiciones de amplificación fueron estandarizadas a partir de lo sugerido por el fabricante del kit. Los genes *babA2* (babA2-F*-* CCAAACGAAACAAAAAGCGT; babA2-R-GCTTGTGTAAAAGCCGTCGT) y *sabA* (sabA-F- TTTTTGTCAGCTACGCGTTC; sabA-R-ACCGAAGTGATAACGGCTTG) se evaluaron mediante reacción en cadena de polimerasa (PCR) convencional según lo sugerido por Yadegar *et al.*^9^. Las cepas de *H. pylori* NCTC-11637, NCTC-11638 y el aislado clínico 3062 fueron facilitadas por el Instituto Nacional de Cancerología como controles positivos. Los productos de amplificación fueron teñidos con 3 μL de EZ-visión (AMRESCO®) y se analizaron mediante electroforesis en gel de agarosa al 1,5% a 80 voltios por una hora, visualizados mediante transiluminador UV con una longitud de onda de 254/365 nm y se compararon con controles positivos y negativos. Se realizaron pruebas piloto para verificar la calidad de los reactivos y kits de extracción, además se verificó la calibración previa de los equipos de laboratorio. 

### Variable dependiente e independiente

Como variables dependientes se incluyeron a las lesiones precursoras de malignidad gástrica, que engloba la gastritis atrófica y la metaplasia intestinal, y a los parámetros histológicos, como el grado de infiltración de células polimorfonucleares y el grado de atrofia. Cada una de estas variables histológicas se categorizó en cuatro niveles de severidad: ninguna, leve, moderada y severa. Las variables independientes abarcaron los genotipos de virulencia de *H. pylori*, que incluyeron *vacA*, *cagA*, *babA* y *sabA*, siendo su unidad de medición la presencia (+) o ausencia (-).

### Covariables

Se analizaron otras variables sociodemográficas como la edad en años, que se categorizó en tres grupos (18-40 años, 41-60 años y ≥ 61 años); sexo (hombres y mujeres); ingreso como la remuneración mínima vital (RMV) donde < 1 RMV significa menor a un sueldo mínimo vital y ≥ 1 RMV significa mayor o igual a un sueldo mínimo vital); lugar de procedencia (urbano y rural); nivel educativo de los pacientes (ninguno, primaria, secundaria y educación superior).

### Análisis estadístico

Las variables categóricas fueron tabuladas utilizando frecuencias absolutas y relativas. Se empleó la prueba de chi cuadrado o Fisher para comparar los genotipos de virulencia de *H. pylori* con las LPMG y parámetros histológicos. Se empleó el software SPSS versión 25. Las variables categóricas se presentan como proporciones y frecuencias absolutas. Se usó el coeficiente de correlación de Cramer para determinar el grado de asociación entre los genotipos. Un valor de p<0,05 se consideró significativo.

### Aspectos éticos

Los participantes aceptaron su participación y firmaron el consentimiento informado. La investigación fue aprobada por el Comité de Ética para la Investigación Científica de la Universidad del Cauca. Acta 6.1-1.25/5 del 23 enero del 2022.

## HALLAZGOS

### Características de la población

La presente investigación tuvo la participación de 290 pacientes. Entre las principales características demográficas, destacó que el grupo de edad comprendido entre los 41 y 60 años prevaleció, representando un 45,2% de la muestra. Además, se observó una marcada predominancia del género femenino, que constituyó el 73,8% de los participantes. Es importante mencionar que un notable porcentaje de la población estudiada tenía ingresos mensuales inferiores al salario mínimo, alcanzando un 87,9%. Respecto a la procedencia geográfica, se evidenció que el 61,7% de los pacientes provenían de entornos rurales. En cuanto al nivel educativo, se observó que la educación primaria fue la más común, abarcando al 57,2% de la población (Tabla 1).

**Tabla 1:** Distribución de las características sociodemográficas de la población de estudio.

Características	Total n=290 (%)
Edad	
18-40 años	88 (30,3)
41-60 años	131 (45,2)
≥ 61 años	71 (24,5)
Sexo	
Hombres	76 (26,2)
Mujeres	214 (73,8)
Ingreso	
<1 RMV	255 (87,9)
≥1 RMV	35 (12,1)
Procedencia	
Urbano	111 (38,3)
Rural	179 (61,7)
Nivel Educativo	
Ninguno	6 (2,1)
Primaria	166 (57,2)
Secundaria	70 (24,1)
Educación superior	48 (16,6)

RMV: remuneración mínima vital; < 1 RMV significa menor a un sueldo mínimo vital; ≥ 1 RMV significa mayor o igual a un sueldo mínimo vital

### Genotipos *vacA, cagA, babA y sabA* y lesiones precursoras de malignidad gástrica

De la población total, 217 fueron positivos para *H. pylori* por diagnóstico molecular de los cuales el 63,6% de los sujetos tenía lesiones precursoras de malignidad gástrica (LPMG) y el 36,4% presentaba gastritis crónica (GC). Se encontró que la presencia de los genotipos de virulencia *vacA* s1/m1 y *cagA*+ fueron más frecuentes en los pacientes con LPMG con 86,3% y 68,1%, respectivamente. El genotipo *vacA* s2/m2 fue más frecuente en pacientes con gastritis crónica. La frecuencia de los genotipos *babA2*+ y *sabA*+ fue mayor en la población con LPMG con 68,8% y 55,8%, respectivamente, aunque no hubo diferencias significativas entre los pacientes con GC y los pacientes con LPMG (p>0,05) (Tabla 2). Adicionalmente, se evaluó el grado de asociación de los genes entre sí. Se evidenció una correlación significativa entre la presencia del gen *vacA* y los genes *cagA y sabA* con grado de asociación media (*V*_*vacA/cagA*_ *=* 0,359, *V*_*vacA/sabA*_ =0,332), al igual que entre los genes *sabA*/*babA2* (*V*_*sabA/babA2*_ *=*0,506), sin embargo, no se encontró correlación entre los demás genes (Tabla 3).

**Tabla 2:** Relación de los genotipos *cag*A, *vac*A, *bab*A2 y *sab*A de *H. pylori c*on las lesiones precursoras de malignidad gástrica.

Genotipos	Total n=217 n (%)	GC n=79 n (%)	LPMG n=138 n (%)	Valor de p ^a^
*cagA -*	78 (35,9)	34 (43)	44 (31,9)	0,099
*cagA +*	139 (64,1)	45 (57)	94 (68,1)	
*babA -*	60 (27,6)	17 (21,5)	43 (31,2)	0,127
*babA +*	157 (72,4)	62 (78,5)	95 (68,8)	
*sabA -*	93 (42,9)	32 (40,5)	61 (44,2)	0,596
*sabA +*	124 (57,1)	47 (59,5)	77 (55,8)	
*vacA*				
*s1/m1*	177 (81,6)	58 (73,4)	119 (86,3)	0,110
*s1/m2*	8 (3,7)	4 (5,1)	4 (2,9)	
*s2/m2*	15 (6,9)	9 (11,4)	6 (4,3)	
Coinfección	17 (7,8)	8 (10,1)	9 (6,5)	

a Valor de p calculado con la prueba chi cuadrado.

GC: gastritis crónica. LPMG: lesiones precursoras de malignidad gástrica.

**Tabla 3:** Relación entre los genotipos de *H. pylori.*

Genotipos	Grado de asociación ^a^	Valor de p
*cagA-babA*	0,138	0,428 ^b^
*cagA-sabA*	0,128	0,601 ^b^
*cagA-vacA*	0,359	0,001 ^c^
*sabA-babA*	0,506	0,001 ^b^
*sabA-vacA*	0,332	0,001 ^c^
*babA-vacA*	0,177	0,152 ^c^

a Grado de asociación calculado con el coeficiente de correlación de Cramer.

b Valor de p calculado con la prueba de chi cuadrado.

c Valor de p calculado con la prueba exacta de Fisher.

### Genotipos *vacA, cagA, babA y sabA* y parámetros histológicos

Considerando la importancia de los diferentes genotipos de virulencia de *H. pylori* sobre los parámetros histológicos, se determinó que la combinación alélica del gen *vacA* s1/m1, los genotipos *cagA*+, *babA2*+ y *sabA*+ se encuentran estrechamente relacionados con la actividad inflamatoria, específicamente, con el grado severo de infiltración de células polimorfonucleares (p<0,05). Además, se evaluó la relación entre los genotipos de virulencia bacteriana y el grado de atrofia gástrica, encontrándose una relación significativa entre la presencia del genotipo *cagA*+ y la ocurrencia de atrofia gástrica en su forma más severa (p<0,05). Otros genotipos no mostraron relación con el grado de atrofia (Tabla 4).

**Tabla 4:** Relación entre los genotipos de virulencia de *H. pylori* y los parámetros histológicos.

Parámetros	Infiltración células polimorfonucleares					Atrofia				
	Ninguna n=52 n (%)	Leve n=41 n (%)	Moderada n=64 n (%)	Severa n=60 n (%)	Valor de p	Ninguna n=81 n (%)	Leve n=51 n (%)	Moderada n=59 n (%)	Severa n=26 n (%)	Valor de p
*cagA-*	29 (55,8)	14 (34,1)	9 (14,1)	26 (43,3)	0,001 ^a^	34 (42,0)	8 (15,7)	24 (40,7)	12 (46,2)	0,007 ^a^
*cagA+*	23 (44,2)	27 (65,9)	55 (85,9)	34 (56,7)		47 (58,0)	43 (84,3)	35 (59,3)	14 (53,8)	
*babA2-*	43 (82,7)	4 (9,8)	6 (9,4)	7 (11,7)	0,001 ^a^	18 (22,2)	15 (29,4)	23 (39)	4 (15,4)	0,071 ^a^
*babA2+*	9 (17,3)	37 (90,2)	58 (90,6)	53 (88,3)		63 (77,8)	36 (70,6)	36 (61)	22 (84,6)	
*sabA-*	49 (94,2)	5 (12,2)	14 (21,9)	25 (41,7)	0,001 ^a^	33 (40,7)	19 (37,3)	33 (55,9)	8 (30,8)	0,091 ^a^
*sabA+*	3 (5,8)	36 (87,8)	50 (78,1)	35 (58,3)		48 (59,3)	32 (62,7)	26 (44,1)	18 (69,2)	
s1/m1	40 (76,9)	28 (68,3)	58 (90,6)	51 (85)	0,001 ^b^	60 (74,1)	44 (86,3)	53 (89,8)	20 (76,9)	0,055 ^b^
s1/m2	0 (0,0)	4 (9,8)	1 (1,6)	3 (5)		4 (4,9)	1 (2,0)	1 (1,7)	2 (7,7)	
Coinfección	11 (21,2)	1 (2,4)	1 (1,6)	4 (6,7)		8 (9,9)	1 (2,0)	4 (6,8)	4 (15,4)	
s2/m2	1 (1,9)	8 (19,5)	4 (6,2)	2 (3,3)		9 (11,1)	5 (9,7)	1 (1,7)	0	

a Valor de p calculado con la prueba de chi cuadrado.

b Valor de p calculado con la prueba exacta de Fisher.

## DISCUSIÓN

En este estudio analizamos la presencia de los genotipos *vacA*, *cagA*, *babA* y *sabA* de *Helicobacter pylori* y su asociación con las lesiones precursoras de malignidad gástrica y parámetros histológicos en pacientes infectados del suroccidente de Colombia. Se encontró que los genotipos s1/m1 del gen *vacA* y *cagA*+ fueron más frecuente en los pacientes con LPMG, siendo similar a lo reportado por estudios previos en Colombia ^10^^,^^11^. Este hallazgo sugiere que en la población de estudio existe una alta frecuencia de cepas bacterianas más virulentas que podrían aumentar el riesgo de enfermedades gastrointestinales. En otras partes del mundo, la distribución de los alelos s1 y m1 se ha asociado con enfermedades como gastritis atrófica, metaplasia intestinal y alto riesgo de cáncer gástrico ^12^. Se encontró una relación entre los genes de virulencia *vacA* con los genes *cagA* y *sabA*, lo que sugiere que estos genes podrían actuar sinérgicamente para aumentar la inflamación gástrica y el riesgo de desarrollar LPMG. El estudio actual encontró una frecuencia mayor de los genotipos *babA2*+ y *sabA*+ en LPMG, aunque no hubo una asociación significativa. Sin embargo, algunos estudios en otros países han descrito que las cepas *babA2*+ y *sabA*+ se relacionan con mayor riesgo de desarrollar atrofia, metaplasia intestinal y cáncer gástrico ^13^^,^^14^. Las diferencias entre poblaciones pueden deberse al pequeño tamaño de la muestra para cada población, a la heterogeneidad entre los estudios y a factores geográficos.

Este estudio es el más reciente en explorar la relación entre los genotipos de virulencia de *H. pylori* y los parámetros histológicos en una población colombiana. Un reporte previo en Colombia señaló que las cepas con los genotipos *vacA* s1 y m1 están estrechamente asociadas con cambios en la histología gástrica, mientras que la infiltración de neutrófilos, la metaplasia intestinal y la atrofia son significativamente mayores en casos positivos para el gen *babA2* en comparación con los casos *babA2* negativos ^15^. En otros estudios realizados en diferentes países, se ha encontrado que la actividad inflamatoria es más pronunciada en pacientes infectados con cepas que albergan todas las variantes virulentas de *H. pylori* (*vacA* s1m1/*cagA*+/*babA2*+) ^16^. También, se ha encontrado que la colonización con factores de virulencia como *cagA*+, *iceA1*- y *oipA*+ se correlaciona significativamente con una alta infiltración de células polimorfonucleares, mientras que la colonización con *cagA*+, *babA2*+ y *oipA*+ está fuertemente relacionada con puntuaciones más altas de infiltración de células mononucleares ^1^. Los anteriores hallazgos son consistentes con lo encontrado en nuestro estudio.

La infección por *H. pylori* causa una inflamación local en la mucosa gástrica debido a la migración e infiltración de células inmunitarias, lo que conduce a la progresión de la enfermedad. Las proteínas *cagA* y *vacA* producidas por *H. pylori* estimulan una respuesta proinflamatoria que conduce a la inflamación gástrica. La producción de IL-8 atrae células inmunitarias para infiltrarse en el sitio de la infección, lo que favorece un estado inflamatorio persistente ^7^^,^^17^. Este hecho puede explicar porque en nuestro estudio el estado positivo de *cagA* y *vacA* se relaciona con la infiltración de células polimorfonucleares. El genotipo *sabA+* también está relacionado con la inducción de la respuesta inflamatoria que conduce al desarrollo de enfermedades gástricas. Las cepas de *H. pylori sabA* positivas estimulan la activación de neutrófilos causando daño epitelial gástrico ^5^. En nuestra población de estudio se encontró una alta infiltración de células polimorfonucleares en pacientes con el genotipo *sabA*+. Estos hallazgos son consistentes con otros estudios que muestran la relación entre la expresión *sabA* y la inflamación gástrica ^18^.

Al analizar los genes de adhesión *babA* y *sabA*, así como los genes *vacA* y *cagA*, podemos ampliar la evidencia sobre su papel en la colonización inicial, la inducción de respuestas inmunológicas y la posterior progresión de lesiones gástricas. Esta investigación contribuirá a llenar el vacío de conocimiento en la población colombiana y puede tener un impacto significativo en la salud pública al identificar subgrupos de pacientes con mayor riesgo de desarrollar enfermedades gástricas graves. Los resultados de este estudio podrían proporcionar información valiosa para orientar estrategias de prevención, diagnóstico temprano y tratamiento efectivo en pacientes con infección por *H. pylori* en Colombia y, posiblemente, en otras poblaciones con características genéticas similares.

Las limitaciones del presente estudio radican en el número pequeño de pacientes involucrados. Además, no se investigaron otros factores de virulencia de *H. pylori* que podrían estar ejerciendo un rol en mecanismos asociados con el desarrollo de lesiones precursoras de malignidad gástrica.

En conclusión, nuestros hallazgos muestran una alta frecuencia del genotipo *vacA* s1/m1 de *H. pylori* en una población colombiana y la actividad citotóxica de este genotipo se podría potenciar con otros factores bacterianos de *H. pylori* como la presencia de los genotipos *cagA+ y sabA+*. La combinación alélica vacA s1/m1 se relaciona con otros factores bacterianos de *H. pylori* como la presencia de los genotipos *cagA*+ y *sabA*+. Este estudio muestra una relación entre los genotipos de virulencia estudiados y el grado de infiltración de células polimorfonucleares, las cuales juegan un papel importante en la actividad inflamatoria, lo que podría incrementar la progresión de lesiones precursoras de malignidad gástrica. Se necesitan más estudios complementarios, incluyendo controles negativos de *H. pylori* para fortalecer esta hipótesis.
